# Identification of protein secretion systems and novel secreted proteins in *Rhizobium leguminosarum *bv. *viciae*

**DOI:** 10.1186/1471-2164-9-55

**Published:** 2008-01-29

**Authors:** Martin Krehenbrink, J Allan Downie

**Affiliations:** 1 Department of Molecular Microbiology, John Innes Centre, Norwich Research Park, Colney Lane, Norwich NR4 7UH, UK; 2 Current address: Unité de Génétique Moléculaire, Institut Pasteur 25, rue du Dr. Roux, 75724 Paris Cedex 15, France

## Abstract

**Background:**

Proteins secreted by bacteria play an important role in infection of eukaryotic hosts. Rhizobia infect the roots of leguminous plants and establish a mutually beneficial symbiosis. Proteins secreted during the infection process by some rhizobial strains can influence infection and modify the plant defence signalling pathways. The aim of this study was to systematically analyse protein secretion in the recently sequenced strain *Rhizobium leguminosarum *bv. *viciae *3841.

**Results:**

Similarity searches using defined protein secretion systems from other Gram-negative bacteria as query sequences revealed that *R. l*. bv. *viciae *3841 has ten putative protein secretion systems. These are the general export pathway (GEP), a twin-arginine translocase (TAT) secretion system, four separate Type I systems, one putative Type IV system and three Type V autotransporters. Mutations in genes encoding each of these (except the GEP) were generated, but only mutations affecting the PrsDE (Type I) and TAT systems were observed to affect the growth phenotype and the profile of proteins in the culture supernatant. Bioinformatic analysis and mass fingerprinting of tryptic fragments of culture supernatant proteins identified 14 putative Type I substrates, 12 of which are secreted via the PrsDE, secretion system. The TAT mutant was defective for the symbiosis, forming nodules incapable of nitrogen fixation.

**Conclusion:**

None of the *R. l*. bv. *viciae *3841 protein secretion systems putatively involved in the secretion of proteins to the extracellular space (Type I, Type IV, Type V) is required for establishing the symbiosis with legumes. The PrsDE (Type I) system was shown to be the major route of protein secretion in non-symbiotic cells and to secrete proteins of widely varied size and predicted function. This is in contrast to many Type I systems from other bacteria, which typically secrete specific substrates encoded by genes often localised in close proximity to the genes encoding the secretion system itself.

## Background

*Rhizobium leguminosarum *bv. *viciae *is a Gram-negative soil bacterium which forms a mutualistic symbiosis with legumes, resulting in nitrogen-fixing root nodules. This symbiotic relationship is initiated by an exchange of signals between the two partners. While the general features of this signal exchange are common to all rhizobia-plant symbioses, differences in the signalling molecules allow only certain bacterium-plant combinations to lead to a successful symbiosis. Plant-made flavonoids released into the rhizosphere [1,2] induce rhizobia to make specific signalling molecules known as Nod factors, which are four or five *β*-1,4 linked *N*-acetyl-glucosamine residues with a fatty acid residue replacing the *N*-acetyl group at the non-reducing end. This basic structure can carry various substituents (such as acetyl, methyl, carbamoyl, sulfuryl and various glycosyl groups) depending on the rhizobial strain, and many strains synthesise a limited variety of Nod factors [3,4].

In addition to Nod factors, secreted proteins can contribute to nodulation ability. NodO is a protein secreted via a Type I secretion system (PrsDE) by some rhizobia and heterologous expression of *nodO *could extend the host range of some rhizobial strains [5,6]. Type I secretion systems transport proteins from the cytoplasm across both membranes to the extracellular space and are usually composed of three gene products: an ATPase of the ATP-binding cassette (ABC) protein family; a membrane fusion protein which spans the periplasm and links the inner and outer membranes [7]; and an outer membrane protein [8]. Their substrate proteins usually carry tandem nonapeptide glycine-rich repeats known as RTX (repeat in toxin) motifs which form a *β*-roll structure stabilised by coordinated Ca^2+ ^ions [9,10]. In *R. leguminosarum *and some other rhizobia, both glycanases involved in bacterial exopolysaccharide (EPS) processing and rhizobial adhesion proteins are secreted via a Type I secretion system (PrsDE) in addition to NodO [6,11-16]. Secreted cell-wall degrading enzymes may help to erode plant cell walls enhancing infection and in *Rhizobium leguminosarum *biovars *trifolii *and *viciae*, cell-associated pectinolytic and cellulolytic enzymes have been found [12,13,17]. *Sinorhizobium meliloti *has been reported to induce the production of polygalacturonase in alfalfa roots [18].

Other rhizobia make use of Type III and Type IV protein secretion systems to inject effector proteins directly into the host plant cells where they modify plant signalling pathways [19-22]. Type III secretion systems translocate proteins across both membranes using ATP. They are assembled from over 20 proteins, many of which closely resemble proteins involved in flagellar biogenesis [23,24]. In some cases, the flagellar apparatus has been shown to export proteins not related to the flagellum [25]. The proteins are normally delivered into the eukaryotic cytoplasm, where they then alter the host metabolism to suit the bacterial needs [26]. In *Rhizobium *sp. NGR234 several proteins including NopX, NopA, NopB, NopP and NopL, were shown to be secreted via a Type III system upon flavonoid induction [21,27-29].

The Vir system of *Agrobacterium tumefaciens *is a well-known Type IV secretion system involved in the transfer of DNA and protein to the plant cell. The Type IV secretory machinery is encoded by the *virB1–11 *genes [30]. A related system in *Mesorhizobium loti *delivers effector proteins into plants during nodule initiation [20].

The twin arginine translocase pathway (TAT) pathway exports folded proteins [31,32] often with posttranslational modifications [31,33-35] and does not recognise unfolded proteins [36]. TAT substrates carry an N-terminal tripartite signal sequence similar to that of GEP substrates, except that the signal peptide is usually longer and carries two consecutive arginine residues near the N-terminus [33,37]. Inactivation of this protein secretion system in *R. l. *bv *viciae *UPM791 led to the inability of the rhizobia to progress beyond the infection zone and the formation of nodules that were unable to fix nitrogen [38].

Several types of protein secretion system function in Gram-negative bacteria, but have not been studied in rhizobia with regard to nodulation. The general export pathway (GEP) is the main pathway for the secretion of proteins to the periplasm in most bacteria. An N-terminal signal peptide which is cleaved from the exported protein by a specific signal peptidase [39]. In *E. coli*, the GEP is composed of a complex of SecD, SecE, SecF, SecG and SecY in the cytoplasmic membrane. In the cytoplasm SecB keeps proteins destined for secretion in an unfolded state [40] and SecA binds to the signal peptide and uses ATP to drive protein export through the SecYEG channel [41-43].

Type II secretion systems translocate proteins from the periplasm and are thus dependent on the GEP or TAT pathways [44,45]. Their substrates consist of folded proteins, and between 12 and 15 different proteins are involved in Type II secretion, many of them sharing features with the Type IV pilus biogenesis system [46].

In Type V secretion systems (also termed autotransporters), the secreted protein transports itself across the outer membrane after being transported to the periplasm by the GEP. Autotransporters have a GEP signal peptide, a variable passenger domain and a C-terminal *β*-domain, which forms a pore in the outer membrane through which the passenger domain passes. The passenger domain is then released by a peptolytic activity or remains covalently bound to the *β*-domain [47]. Many autotransporter proteins have an unusually long N-terminal signal peptide of more than 42 amino acid residues [47,48]. The so-called unlinked autotransporters or two-partner secretion (TPS) systems [49,50] have separate passenger and *β*-domain proteins, each carrying a GEP signal peptide. Export of the passenger domain from the periplasm proceeds via an outer membrane pore formed by the *β*-domain.

Fimbriae and Pili are not commonly regarded as protein secretion systems, but do transport the pilus or fimbrial subunit proteins across the outer membrane. The subunit proteins are usually exported to the periplasm by the GEP, and some proteins involved in pilus biosynthesis display similarity to components of the Type II protein secretion machinery. In Pap fimbriae, the fimbrial usher proteins mediate the assembly of the fimbrial subunits and also form a pore through which the fimbriae pass through the outer membrane.

MscL and holin both form channels within the inner membrane through which proteins can pass under certain conditions. Holins are often phage-derived and facilitate the export of autolysin proteins leading to the degradation of the cell wall and death. The pores formed by MscL only open during severe hypoosmotic shock and are though to be primarily involved in rapidly relieving excessive turgor by allowing low- to medium-molecular weight osmolytes to diffuse out of the cell. The channels do however also open wide enough to allow the escape of small proteins such as thioredoxin into the periplasm [51,52].

In this study, the genome of *R. l. *bv. *viciae *3841 was analysed for the presence of known protein secretion systems to assess their role in the nodulation process.

## Results

### Identification of genes encoding different protein transport systems

#### a) Identification of general export pathway (GEP) genes

The components of the GEP from *Escherichia coli *were used as query sequences to search the *R. l*. bv. *viciae *3841 genome database using the BLASTP algorithm [53]. *R. l*. bv. *viciae *3841 was found to carry single genes coding for SecA (RL4298), SecB (RL0006), SecD (RL0680), SecE (RL1759), SecF (RL2055), SecG (RL2512) and SecY (RL1794), which suffice to constitute a functioning GEP machinery (Table 1). These chromosomal genes were not located in operons or even close to one another. Only one copy of each of the genes encoding type I (RL1510) and type II (RL0410) signal peptidases were found in the *R. l*. bv. *viciae *3841 genome.

**Table 1:** Homologs of known protein secretion systems identified in the genome of *R. l*. bv. *viciae *3841.

**System**	**Homolog I**	**Homolog II**	**Homolog III**	**Homolog IV**
*a) General export pathway*				
SecB	RL0006			
SPase II	RL0410			
YidC	RL0453			
SecD	RL0680			
SPase I	RL1510			
SecE	RL1759			
SecY	RL1794			
SecF	RL2055			
SecG	RL2512			
SecA	RL4298			
FtsY	RL4543			
Ffh	RL4547			
*b) Twin arginine pathway*				
TatA	RL2046			
TatB	RL2047			
TatC	RL2048			
*c) Type I pathway*				
AprE	RL3657	RL0071	RL0622	pRL90165
AprD	RL3658	RL0072	RL0623	pRL90164
TolC	none			
*d) Type II pathway*				
Any component	none			
*e) Type III/flagellar pathway*				
FliF	RL0695			
FlhB	RL0699			
FliG	RL0700			
FliN	RL0701			
FliM	RL0702			
FliI	RL0705			
FlgI	RL0712			
FlgH	RL0714			
FliL	RL0715			
FliP	RL0716			
FliQ	RL0734			
FlhA	RL0735			
FliR	RL0736			
*f) Type IV pathway*				
VirB1	pRL70149			
VirB2	pRL70150			
VirB3	pRL70151			
VirB4	pRL70152			
VirB5	pRL70153			
VirB6	pRL70154			
VirB8	pRL70155			
VirB9	pRL70156			
VirB10	pRL70157			
VirB11	pRL70158			
*g) Type V pathway*				
AT	RL1927	RL1196	RL1069	
TPS	None			
*h) Holins*				
Holin	None			
*i) Large conductance mechanosensitive channel*				
MscL	RL0602			
*j) Pilus*				
PilQ	RL0210			
PilA	RL0211			
CpaA	RL0212			
CpaB	RL0213			
CpaC	RL0214			
CpaD	RL0215			
CpaE	RL0216			
CpaF	RL0217			
TadB	RL0218			
TadC	RL0219			
*k) Fimbrial usher protein*				
PapC	none			

#### b) The *tatABC* genes of the twin-arginine translocation pathway (TAT)

To search for components of the TAT machinery, the sequences of TatA, TatB, TatC and TatE from *E. coli *were used as queries. No copy of *tatE *was found, but *tatA *(RL2046), *tatB *(RL2047) and *tatC *(RL2048) appeared to be located within one operon. The same organisation is found *A. tumefaciens *[54] and *R. l*. bv. *viciae *UPM 791 [38] and in the published genome sequences of other rhizobia such as *R. etli *CFN 42 [55] and *S. meliloti *1021 [56].

#### c) Identification of genes encoding four predicted Type I protein secretion systems

**Figure 1:**
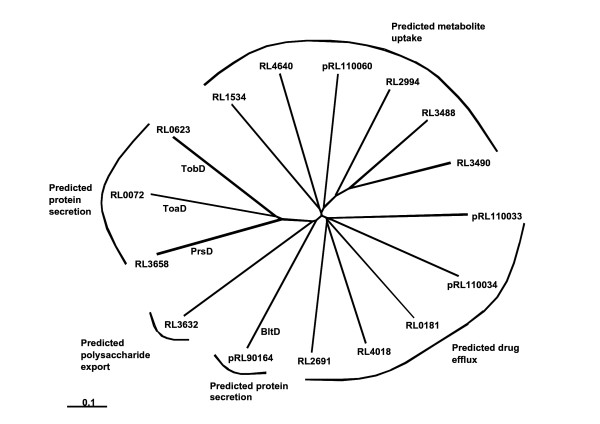
**Unrooted dendrogram of *R. l*. bv *viciae *3841 proteins with similarity to PrsD**. The bar represents 0.1 amino acid changes; predicted functions for proteins are given.

The *prsDE *genes encode a typical Type I secretion system in rhizobia [12]. Using PrsD and PrsE as queries, three novel putative Type I systems were identified in addition to PrsDE. As the search was expected to return many putative ABC transporters not related to protein secretion [57], the 11 closest hits to the PrsD sequence were queried against the *R. l*. bv. *viciae *3841 genome and pooled with the 11 closest hits for each query to give a subset of 16 unique putative protein sequences (most of the 11 × 11 = 121 sequences were repeat hits within the sets of 11). These 16 sequences were then queried against the NCBI database using BLASTP [53]. This revealed that (a) three sequences (including PrsD) were most similar to protein transporters of the AprD-type (alkaline protease) from *Pseudomonas *sp., (b) one was most similar to bacteriocin/lantibiotic transporters (SunT-type), and (c) the rest were most similar to ABC drug/metabolite transporters and polysaccharide exporters. A radial tree (Fig. 1) shows the relationships of the 16 identified ABC proteins. The four ABC proteins predicted to be involved in protein secretion all contained a conserved ATP-binding Walker A region (GXXGXGKT/S) [58] and are the PrsDE system, two predicted PrsDE-like systems encoded by genes we have named the *toaDE *(RL0072 and RL0071) and *tobDE *(RL0623 and RL0622) genes and the *bltDE *genes (pRL90164 and pRL90165) encoding putative proteins with similarity to bacteriocin/lantibiotic exporters. The other proteins with the highest similarity to ABC drug/metabolite transporters and polysaccharide exporters are thought to be involved in the transport of small molecules or polysaccharides rather than proteins.

#### d) Identification of predicted Type I substrates

Genes encoding Type I substrates are often close to the genes encoding Type I secretion systems. The *prsDE *genes are preceded by *plyA *encoding the cell-bound glycanase PlyA, a known substrate of the PrsDE system [13,59]. None of the genes within 10 kb of the *toaDE *(RL0072/RL0071) or *tobDE *(RL0623/RL0624) encodes proteins which are known Type I substrates.

Proteins secreted by Type I secretion systems often contain repeated acidic RTX (repeat in toxin) motifs probably involved in protein folding in the extracellular space [10,60]. The known Type I proteins with RTX domains, NodO from *R. l*. bv. *viciae *8401/pRL1 [GenPept:P15728], HlyA from *E. coli *O157:H7 [GenPept:AAA20544], and LipA from *Serratia marcescens *[GenPept: BAA02519], were used as to search the *R. l*. bv. *viciae *3841 database using BLASTP. The resulting 15 highest-scoring proteins were each used to search the non-redundant NCBI database for similar proteins (the 15 highest-scoring proteins were chosen as a cut-off instead of a defined *expect*-value to accommodate for proteins of varying lengths). If the highest similarity was to a known non-substrate, the protein was added to a list of false positives. Proteins with high similarity to known secreted proteins were listed as possible true positives. Any new proteins from *R l*. bv. *viciae *3841 identified as possible Type I substrates were re-tested as query sequences for the *R l*. bv. *viciae *3841 genome and the whole process reiterated until it converged on a defined set of sequences. Every protein sequence within the set could be used as an initial query to return all members of the set. These sequences were additionally analysed for the presence of repeat sequences using the DOTTER software [61].

Using this approach, 14 different proteins predicted to be secreted by a Type I secretion system were identified. Seven were already known to be secreted via the PrsDE system in *R. leguminosarum *or were very similar to known substrates [12,13,62]. Of these seven, three were rhizobial adhering proteins (RapA2, RapB and RapC), two were predicted glycanases (PlyA and PlyB), one was the nodulation protein NodO, and one (RL3023) was 63% identical to PlyB and so was named PlyC.

**Figure 2:**
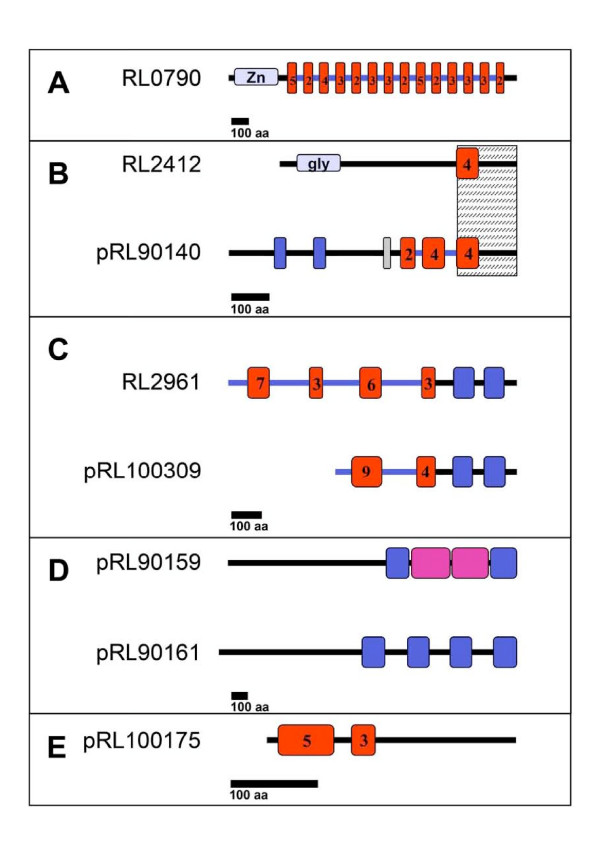
**Domains found in proteins prediced to be secreted via a Type I system**. Coloured elements represent repeated sequences. Red denotes clusters of repeated RTX nonapeptides; the numbers of repeats are indicated. A: Zn, Zn-metalloprotease domain. B: Blue boxes represent single repeated domains; gly, glycosyl hydrolase domain; dark grey, TPT repeat; hatched area marks sequence similarity. C: Blue boxes represent single cadherin domains. D:. Blue, repeated sequence 1; magenta, repeated sequence 2. E: NodO protein shown for comparison. Bar represents 100 amino acid residues.

The sequence motif structures of the other seven predicted Type I secretion substrates are summarised in Fig. 2, including NodO for comparison. Of these RL0790 was by far the most strongly predicted new Type I substrate. While the N-terminal 250 amino acid residues had similarity (32% identity) to the Zn-dependent metalloprotease PrtA from *Pseudomonas fluorescens *SIK W1 [GenPept:AAD09851], most of the protein was made up of an extensive RTX motif region carrying a total of 80 nonapeptide motifs occuring in 14 clusters of 2–5 units repeated roughly every 40 amino acids (Fig. 2A). These 40 amino acids constitute a repeated domain that occurs 13 times. The ~50 C-terminal residues are not part of a repeat.

RL2412 and pRL90140 were very similar (76% identity) in their last 150 amino acid residues, but completely different in the rest of their sequences (Fig. 2B). RL2412 carries one region of four consecutive RTX motifs towards the C-terminus and has limited similarity to glycosyl hydrolases in the N-terminal region (CD-score 31 bits (70) in conserved domain database RPSBLAST search). In pRL90140, there are three RTX motif regions containing 2, 4 and 4 RTX motifs respectively. It contains the sequence Thr-Pro-Thr repeated in tandem six times (TPT TPT TPT TPT TPT TPT) just in front of the first RTX region. The N-terminus contains two copies of a 32 amino acid sequence (41% identity to each other) that resembles repeats in cyanobacterial parallel beta-helix repeat proteins of unknown function (56% and 53% identity to a protein from *Anabaena variabilis *ATCC 29413 [GenPept:YP_322399] for the first and second repeat, respectively).

RL2961 and pRL100309 (Fig. 2C) are similar, but differ in the number of RTX motifs towards the N-terminus (four in RL2961, two in pRL100309). Both contain putative calmodulin-like calcium-binding motifs repeated two times towards the C-terminus. Similar motifs are found in hypothetical proteins from other bacteria (e.g. amb3275 from *Magnetospirillum magneticum *AMB-1 [GenPept:YP_422638] and bll3714 from *Bradyrhizobium japonicum *USDA 110 [GenPept:BAC48979], 39% identity in both) and also resemble cadherins, calcium-binding cell-cell adhesion proteins from animal cells (35% identity to mouse cadherin-10, [GenPept: AAH62962]). Cadherins are involved in mediating the formation of the adherens junction in animals in a calcium-dependent manner [63].

The genes encoding pRL90159 and pRL90161 are both located just upstream of the putative bacteriocin/lantibiotic transporter system *bltDE*. Both encode similar, very large proteins with predicted sizes of 179 kDa and 184 kDa, respectively. They lack a classical RTX domain, but do contain a repeated ~140 amino acid motif of unknown function. These motifs are repeated four times in the C-terminal half of pRL90161, two times in pRL90159 (Fig. 2D) and display 55–65% identity to four similar repeats in a hypothetical protein from *Nitrobacter hamburgensis *X14 [GenPept:YP_578257]. In pRL90159, the second and third repeats are replaced by two copies of a 220 amino acid sequence (96% identity to each other) of unknown function (Fig. 2E).

#### e) Type II and Type III protein secretion systems are absent, but a Type IV system may be present

Although Type II secretion systems are common and widespread in many Gram-negative bacteria such as *Pseudomonas *spp. [64], no components of this pathway were found in *R. l*. bv. *viciae *3841 using PulC-O from *Klebsiella oxytoca *as query sequences. The only proteins with similarity to components of Type III secretion systems were components of the flagellar apparatus.

Using the VirB1–11 proteins of *A. tumefaciens *as a query, two separate clusters of genes were found. The first is on the transmissible plasmid pRL8 and is very similar to other plasmid transfer systems (*trb*) implying that these genes are most likely to be involved in plasmid transfer, rather than protein secretion. The second cluster is on the transmissible plasmid pRL7 and contains putative *virB1, virB2*, *virB3*, *virB4*, *virB5*, *virB6, virB8*, *virB9*, *virB10 *and *virB11 *genes (pRL70149–pRL70158), thus possessing essential genes for DNA and/or protein transport via a Type IV secretion system [65]. The presence of this cluster does however not necessarily identify this cluster as a protein secretion system. As the process of type IV protein export is not yet well understood, it is not possible to tell from sequence data alone whether these components are involved in protein export or conjugal DNA transport.

#### f) Identification of genes encoding Type V systems

Three putative proteins with sequence similarity to the autotransporter BrkA [GenPept:AAA51646] from *Bordetella pertussis *were identified from the *R. l*. bv. *viciae *3841 genome (Table 1). The amino acid sequences of all three putative autotransporters included predicted N-terminal signal peptides of 35 (AutA, RL1927), 41 (AutB, RL1196) and 29 (AutC, RL1069) amino acid residues as predicted by SignalP 3.0, Hidden Markov model [66]. While the *β*-domain was well conserved in all three cases, no significant similarity to any known or putative protein was found for the N-proximal passenger domain. No members of a two-partner secretion system were detected using the *β*-domains of BrkA and the identified putative autotransporters as query sequences.

The three predicted autotransporter proteins encoded by *autA*, *autB *and *autC *in different regions of the chromosome are large (1290, 875 and 1204 amino acid residues, respectively). The 300–450 C-terminal amino acid residues of each could be assigned to the autotransporter *β*-domain by similarity to the *β*-domains of known autotransporters including BrkA. As the *β*-domain of autotransporters is well conserved across species, it was omitted from BLASTP searches to avoid skewing the results towards proteins with unrelated passenger domains. The protein most similar to the passenger domain of AutA was a predicted autotransporter [GenPept:YP_296187] from *Ralstonia eutropha *JMP134 (36% identity). AutB was most similar to a predicted autotransporter [GenPept:YP_673151] from *Mesorhizobium *sp. BNC1 (43% identity). The protein most similar to the passenger domain of AutC was a putative autotransporter [GenPept:YP_485594] from *Rhodopseudomonas palustris *HaA2 (33% identity). There was no significant similarity to the passenger domains of autotransporters from other rhizobia. The genetic neighbourhood of the genes also did not yield any direct indication to possible functions.

#### g) Identification of genes encoding fimbriae, pili, MscL channels and holins

Although no fibrial usher proteins were found, the *R. l*. bv. *viciae *3841 genome contains a cluster of genes predicted to be involved in Cpa pilus biogenesis (Table 1). The *R. l*. bv. *viciae *3841 genome was found to encode no holins and one putative MscL protein (RL0602).

### Phenotypes of mutants affected in predicted secretion systems

To determine the functions of the identified putative protein secretion systems, we generated derivatives of strain 3841 carrying insertion mutations in *prsD *(A895), *toaD *(A913), *tobD *(A896), *virB6 *(A897), *autA *(A1010), *autB *(A1011), *autC *(A1012) and *tatC *(A1052). The mutants were tested for polygalacturonase (PGase) activity, glycanase activity, motility, biofilm formation and growth on different carbon sources (mannitol and succinate). All mutants were able to grow on TY, Y-succinate and Y-mannitol plates.

None of the mutants was affected in cell-associated PGase activity, suggesting that this activity is independent of the identified secretion systems. The *prsD *(A898) mutant lacked extracellular glycanase activity, failed to form biofilm rings in shaking culture, and produced very mucoid colonies and viscous culture supernatants (not shown). Such phenotypes were observed previously with a *prsD *mutant of *R. l*. bv. *viciae *strain A34 [12,59]. No such effects were observed with any of the other mutants including A913 (*toaD*), or A896 (*tobD*). To rule out the possibility of additive phenotypes or redundancy between the different Type I systems, we constructed a *toaD/tobD/prsD *triple mutant (A912) and *toaD/prsD *(A911) and *tobD/prsD *(A903) double mutants, but all had the same phenotypes as A898 (*prsD*).

Whereas the wild type (and all the other mutants tested) swarmed outward from the point of inoculation on 0.2% agar TY plates, the *tatC *mutant (A1052) formed a tight colony and showed no sign of swarming behaviour. Growth of the *tatC *mutant in liquid TY medium was slightly impaired (it had a doubling time of 4.5 h compared with 3 h for WT and the other mutants). Microscopy revealed that the *tatC *mutant was much more rounded in shape than the wild type.

### All mutants except A1052 (*tatC*) are capable of effective nodulation

**Figure 3:**
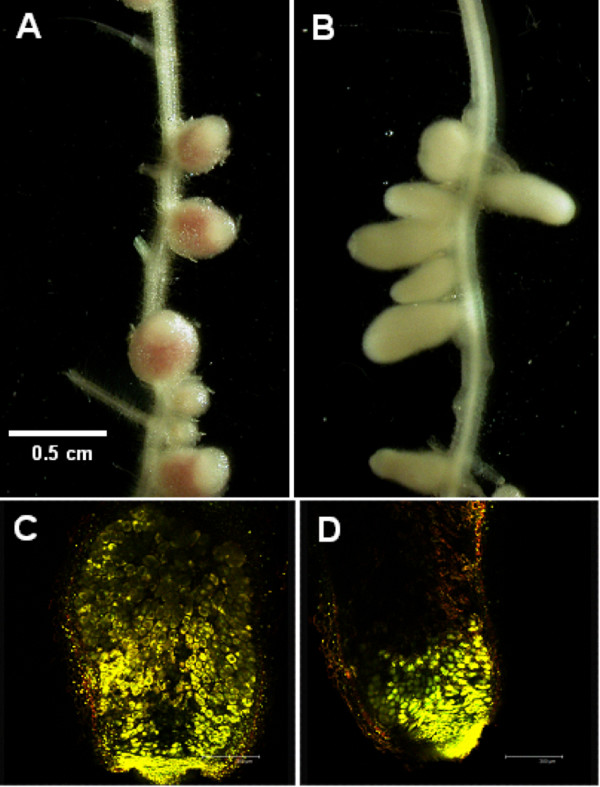
**The *R. l*. bv. *viciae *3841 *tatC *mutant exhibits anomalous infection of pea nodules**. Nodules formed on pea plant roots (cv. Frisson) three weeks after inoculation with wild type *R. l*. bv. *viciae *3841 (A, C) and the *tatC *mutant A1052 (B, D). A and B are images of fresh nodules, C and D were nodules preserved in 70% ethanol before staining with SYTO 13 dye. Structures of high DNA content (bacteria and plant cell nuclei) are stained yellow. The nodule tip is at the bottom.

To examine the role of the identified secretion systems in nodulation, pea seedlings (cv. Frisson) were inoculated with wild type *R. l*. bv. *viciae *3841, A895 (*prsD*), A913 (*toaD*), A896 (*tobD*), A897 (*virB7*), A1010 (*autA*), A1011 (*autB*), A1012 (*autC*) and A1052 (*tatC*). After three weeks growth on nitrogen-limited medium, the plants inoculated with the *tatC *mutant (A1052) looked nitrogen starved, and the nodules formed were unable to fix nitrogen based on measurements of acetylene reduction; staining of these nodules with SYTO 13 [67] revealed bacteria near the tip of the nodule, but they did not extend as far into the nodule as wild type bacteria (Fig. 3). All of the other mutants formed normal numbers of nitrogen-fixing nodules (data not shown). Nodulation experiments carried out with the alternative host plants broad bean (*Vicia faba *cv. The Sutton) and hairy vetch (*Vicia hirsuta*) mirrored the results obtained on pea plants.

### The *tatC *mutant has enhanced levels of proteins in the culture supernatant but lacks flagellin

**Figure 4:**
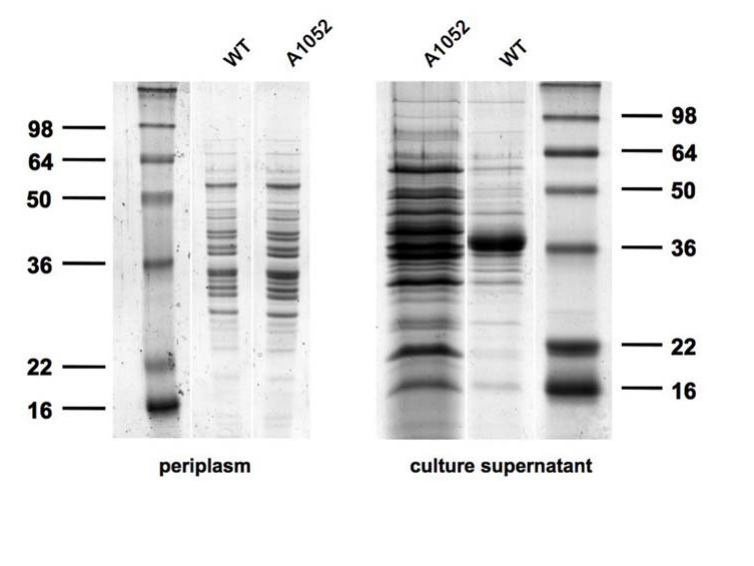
**The *tatC *mutant of *R. l*. bv. *viciae *3841 is altered in the culture supernatant protein profile, but is very similar to the wild type in its periplasmic protein profile**. *R. l*. bv. *viciae *3841 and the *tatC *mutant (A1052) were grown for three days in 100 ml TY (periplasm) or for four days in 100 ml Y-mannitol medium (culture supernatant). Periplasmic proteins were prepared by cold hypo-osmotic shock, ~30 μg were loaded per lane. Culture supernatant proteins were precipitated with TCA and protein representing 20 ml of culture were loaded in each lane.

A comparison by SDS-PAGE of the proteins in the culture supernatant of the *tatC *mutant (A1052) and the wild type grown in Y-mannitol liquid medium showed clear differences in the protein pattern (Fig. 4). Although there were many bands in common, A1052 consistently contained more total protein in the culture supernatant as estimated by loading proteins precipitated from equal amounts of culture supernatant. The abundant proteins of around 36 kDa produced by the wild type but missing from the supernatant of A1052 (*tatC*) were identified by MALDI-ToF as flagellins (RL0718 and RL0720). Although the TAT pathway secretes proteins to the periplasm and not directly to the extracellular space, the absence of flagellar proteins from the culture supernatant of a *tatC *mutant was previously shown for *A. tumefaciens *[54].

Periplasmic proteins from *R. l*. bv. *viciae *3841 and A1052 (*tatC*) were isolated by cold hypo-osmotic shock and analysed by gel electrophoresis. The absence of cytoplasmic contamination of the periplasmic protein preparation was checked by measuring glucose-6-phosphate dehydrogenase (G6PDH) activity [68]. Specific G6PDH activity in the periplasmic preparations was less than 4% of the specific G6PDH activity in whole cell extract. Although the TAT machinery exports proteins to the periplasm, there were no major differences in the periplasmic protein profile of *R. l*. bv. *viciae *3841 and the *tatC *mutant A1052 (Fig. 4.).

### Identification of proteins present in the culture supernatant of mutants in predicted protein secretion systems and the wild type

**Figure 5:**
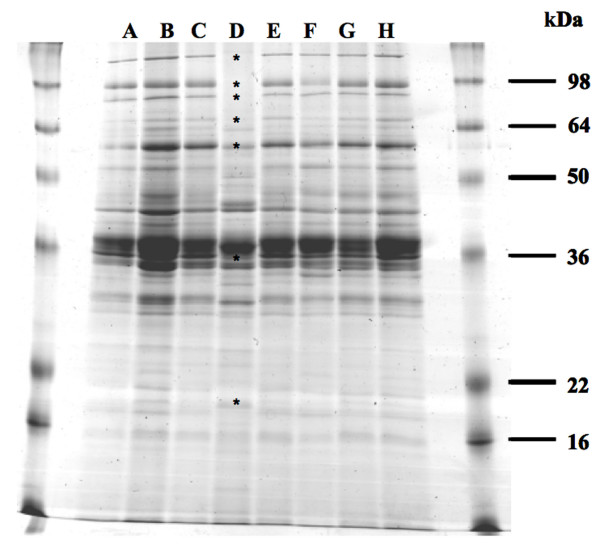
**Culture supernatant proteins from extracellular protein secretion system mutants**. Cells were grown in Y-mannitol medium for 4 days. Proteins were precipitated with trichloroacetic acid. A, *R. l*. bv. *viciae *3841 wild type; B, *toaD *mutant; C, *tobD *mutant; D, *prsD *mutant; E, *virB6 *mutant; F, *autA *mutant; G, *autB *mutant; H, *autC *mutant. Protein bands missing in the lane representing the *prsD *mutant are marked (*).

Culture supernatant proteins of *R. l*. bv. *viciae *3841and the *prsD *(A895), *toaD *(A913), *tobD *(A896), *virB6 *(A897), *autA *(A1010), *autB *(A1011) and *autC *(A1012) mutants grown in Y-mannitol medium were analysed by SDS-PAGE. Only the supernatant protein profile from the *prsD *mutant (A895) differed from that of the wild type and was missing seven protein bands (Fig. 5). The seven proteins corresponding to the indicated bands in Fig. 5 were identified by MALDI-ToF MS fingerprinting (Table 2). Six of these were either known Type I substrates (PlyB and RapA2) [62] or were predicted above (RL2961, pRL100309, pRL90140 and RL2412) to be secreted using bioinformatic analyses.

**Table 2:** Culture supernatant proteins from *R. l*. bv. *viciae *3841 were identified by MALDI-ToF MS.

**Band**	**Apparent weight [kDa]**	**Top MOWSE score**	**Number of matched fragments**	**Predicted protein**	**Theoretical weight [kDa]**	**Predicted function**
*1	89.1	205	18	RL2961	95.0.	cadherin-like protein
*2	59.2	165	15	pRL90140	79.0.	parallel beta-helix repeat protein
*3	57.7	113	15	pRL100309	60.7	cadherin-like protein
*4	52.2	78	8	RL2412	64.6	glycosyl hydrolase
5	50.2	243	20	RL4651	63.7	dipeptide-binding protein
6a	46.5	55	7	RL0778	58.8	dipeptide-binding protein
*6b	46.5	195	16	RL3024	51.6	PlyB
7	41.5	101	10	RL0728	44.4	flagellar hook
8	38.5	174	14	RL4218	47.6	sorbitol-binding protein
9	37.5	76	8	RL3329	43.4	membrane bound lytic tranglycosylase
10	35.6	77	7	RL3745	38.9	Leu/Ile/Val-binding protein (BraC)
11a	34.1	60	6	RL0720	33.6	flagellin
11b	34.1	96	9	RL2375	38.9	basic membrane lipoprotein
*12a	29.8	58	5	pRL100451	24.9	RapA2
12b	29.8	60	6	RL0196	35.0.	basic membrane lipoprotein
12c	29.8	44 (53)	5	RL0489	35.4	sugar-binding protein
12d	29.8	36 (51)	4	RL0718	31.0.	flagellin
13	27.7	129	10	RL0518	39.4	ribose-binding protein
14	25.0.	113	9	RL2753	27.9	arginine/ornithine-binding protein
15	20.4	57	5	RL1369	24.5	pentapeptide repeat protein
16	17.1	85	6	RL2404	20.5	peptidyl prolyl cis-trans isomerase
*17	16.3	83	6	RL1580	15.3	nucleoside diphosphate kinase
18	16.0	70	5	RL2697	19.5	COG3184

**Figure 6:**
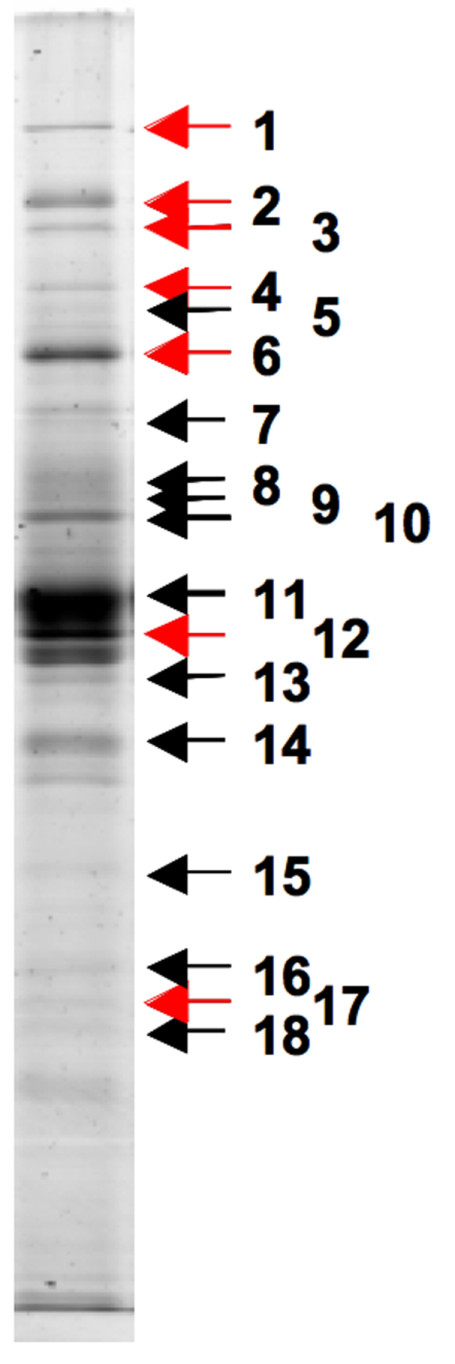
**Protein bands submitted to MALDI-ToF MS analysis**. Supernatant proteins were separated by SDS-PAGE and stained with colloidal Coomassie G. The indicated protein bands present in the wild type were excised and analysed by MALDI-ToF MS fingerprinting. Protein band missing in the culture supernatant of the *prsD *mutant (A898) are indicated by red arrows.

The protein bands labelled in Fig. 6. were cut out from the gel and digested with trypsin. The masses of the tryptic digest were identified by MALDI-ToF MS and searched against a *R. l*. bv. *viciae *3841 genome database. Band #, protein band as labelled in Fig. 6., Band from which more than one protein was identified are indicated (e.g. 6a, 6b). Apparent weight, molecular weight of the proteins in the given band as estimated from the electrophoretic mobility. Top MOWSE score, statistical score calculated by the MASCOT program for the *R. l*. bv. *viciae *3841 indicated in column 'predicted protein', scores above 50 indicate >95% confidence. Number of matched fragments, number of fragment masses which could be assigned to the predicted protein. Predicted protein, proteins scoring more than 50 as MOWSE score. Theoretical weight, calculated molecular weight of the indicated proteins. Scores obtained after refining search parameters are shown in brackets. An asterisk indicates PrsDE-dependence.

One of the proteins missing from the culture supernatant of the *prsD *mutant (A895) was a predicted nucleoside diphosphate kinase (NDK, RL1580). NDK is generally a cytoplasmic enzyme involved in nucleotide metabolism [69] and catalyses the general reaction N_x_TP + N_y_DP ↔ N_x_DP + N_y_TP, where N_x or y _can be any base. Therefore, its presence in the supernatant of the wild type was unusual, although NDK has been shown to be secreted via a Type I system in *P. aeruginosa *[70]. The absence of NDK from the culture supernatant of A898 (*prsD*) indicates specific export by the PrsDE Type I system. To rule out cytoplasmic contamination of the culture supernatant, the small (~22 kDa) and highly expressed cytoplasmic protein RhiA was used as a marker. RhiA is a protein of unknown function [71,72], and its high abundance and the availability of a specific antiserum [73] made it a good choice as a marker protein, because cell lysis would release large amounts of RhiA into the culture medium. RhiA could not be detected in the culture supernatant of bacteria grown for 4 days to mid-stationary phase (data not shown).

Double and triple mutants in *toaD*, *tobD *and *prsD *were made but no effects on supernatant proteins were seen unless the strain also carried a mutation in *prsD*, in which cased the same changes as in a *prsD *single mutant were observed. A mutation in *bltD *similarly had no observed effect on the profile of proteins isolated from the culture supernatant (data not shown).

Although the *prsD *mutant differed from the wild type in culture supernatant protein profile (Fig. 5), this only accounted for seven of the many culture supernatant protein bands observed by SDS-PAGE. Most of these bands were also observed in periplasmic fractions of wild type *R. l*. bv. *viciae *3841, and many were indeed very abundant in this fraction, suggesting that these proteins might have reached the culture supernatant through unspecific leakage. Nevertheless, three protein bands remained which were exclusive to the culture supernatant. To identify these other proteins, bands 7, 11 and 12 were excised from the gel for MALDI-ToF MS analysis. Select bands present in both the culture supernatant and the periplasmic fraction were also excised from the gel to confirm their periplasmic origin. Table 2 and Figure 6 list 23 different proteins (including all seven bands missing from the supernatant of the *prsD *mutant) that were identified from the culture supernatant. Of these, proteins RL0718, RL0720 and RL0728 (Table 2) are predicted to be extracellular components of the flagellum and expected to be present in the culture supernatant [74-76]. Out of the 23 identified supernatant proteins (Table 2), 13 were predicted to be periplasmic by the SignalP 3.0 software [66] (Table 3). The distinction between signal peptide proteins and leaderless proteins is very clear, with proteins either having a signal peptide probability of 0.999 or better or less than 0.033. The signal peptides are predicted to be between 20 and 32 amino acid residues in length with probabilities between 0.803 and 1.000. One of the predicted signal peptide proteins (RL0518) is also predicted to be a TAT substrate by the TATP 1.0 [77], but not by the TATFIND [78] algorithm. The predicted signal peptide of RL0518 carries a twin-arginine motif, and the surrounding residues also suggest a TAT leader peptide. The absence of this protein could not be confirmed in the culture supernatant of the *tatC *mutant A1052 (Fig. 4) this could either be due to the abundance of other proteins in the culture supernatant masking its absence or the possibility that it is not a TAT substrate. Five of the supernatant proteins were predicted to be periplasmic substrate-binding components of ABC uptake transporters, two each were basic membrane proteins or conserved proteins of unknown function and one was predicted to be peptidyl-prolyl cis-trans isomerase. None had similarity to any known extracellular proteins from other bacteria. Table 3 lists all proteins identified in the culture supernatant and gives their predicted function and likely secretion system.

**Table 3:** Prediction of signal peptides of proteins identified in the culture supernatant

**Protein**	**Signal peptide probability**	**Maximum cleavage site probability**	**Predicted signal length (aa)**	**Predicted signal peptide**	**Predicted secretion system**	**Predicted function**
RL0196	1.000	0.999	20	MKKSLLTLFAVAAMSTTALA	GEP	basic membrane lipoprotein
RL0489	1.000	1.000	23	MKKSVLAFGALALGVTFSAPVMA	GEP	sugar-binding protein
RL0518	1.000	1.000	32	MSIVKSLLS**RR**AFTALAGAAVIATAMPAPSFA	TAT	ribose-binding protein
RL0778	1.000	0.956	23	MKKISVLLAATALISVMATSAWS	GEP	dipeptide-binding protein
RL1369	0.999	0.803	25	MAMHGSSLGLLALVLLAFPPVAARA	GEP	pentapeptide repeat protein
RL2375	1.000	0.944	24	MKKLALALAATAALVLSIGSAAEA	GEP	basic membrane lipoprotein
RL2404	1.000	0.983	23	MKLFYLAFAGVLYLASFAGDAFA	GEP	peptidyl prolyl cis-trans isomerase
RL2697	0.999	0.933	28	MMNIAGLGRLAAATVVLSGLAFGSAVKA	GEP	COG3184
RL2753	1.000	1.000	25	MLNSTRIFAAASIAAMSLFAGSAMA	GEP	arginine/ornithine-binding protein
RL3329	1.000	0.975	25	MHRSLASCSALALLFALALAGGAAA	GEP	membrane bound lytic tranglycosylase
RL3745	1.000	0.999	23	MKKSLLSAVALTAMVAFSGNAWA	GEP	Leu/Ile/Val-binding protein (BraC)
RL4218	1.000	0.998	22	MTLRTFLLGACSALAFAGMASA	GEP	sorbitol-binding protein
RL4651	1.000	1.000	29	MTKLNRNFRMLSAGAALSLLMMAAPSAFA	GEP	dipeptide-binding protein

pRL90140	0.000	0.000	-	-	PrsDE	parallel beta-helix repeat protein
pRL100309	0.000	0.000	-	-	PrsDE	cadherin-like protein
pRL100451	0.000	0.000	-	-	PrsDE	RapA2
RL1580	0.000	0.000	-	-	PrsDE	nucleoside diphosphate kinase
RL2412	0.000	0.000	-	-	PrsDE	glycosyl hydrolase
RL2961	0.000	0.000	-	-	PrsDE	cadherin-like protein
RL3024	0.000	0.000	-	-	PrsDE	PlyB

RL0718	0.002	0.001	-	-	Flagellum	flagellin
RL0720	0.012	0.005	-	-	Flagellum	flagellin
RL0728	0.032	0.029	-	-	Flagellum	flagellar hook

Signal peptide prediction was done using the hidden Markov model of the SignalP 3.0 software [66]. Probabilities for the presence of a signal peptide and for the correct identification of the cleavage site are given. The TAT signal prediction software TATP 1.0 predicts the same signal peptide length for the predicted TAT substrate RL0518. The twin-arginine motif of the RL0518 protein is in bold. The prediction of the secretion system involved in the export of the identified proteins was based on signal peptide prediction (GEP and TAT), observed absence from the culture supernatant of the *prsD *mutant (PrsDE) and similarity to known proteins (Flagellum).

## Discussion

### Type I protein secretion in *R.l*. bv. *viciae *3841

*The only Type I secretion systems studied in rhizobia so far have been the PrsDE system *[12,13,62]*and systems dedicated to the export of bacteriocin *[79,80]. *This study reports the presence of a total of *four predicted Type I secretion systems in *R.l*. bv. *viciae *3841, encoded by the *prsDE*, *toaDE, tobDE *and *bltDE *genes. Of these, the *prsDE*-encoded system has been characterised previously [12,13,62] and is conserved in *S. meliloti *[15], *R.l*. bv. *trifolii *[81], *R. etli *and *A. tumefaciens*. Of the fourteen predicted Type I substrates identified in *R.l*. bv. *viciae *3841, seven (NodO, PlyA, PlyB, PlyC, RapA2, RapB and RapC) were already known or predicted to be secreted by the PrsDE system based on previous work [12,13,62,82]. The other seven predicted Type I substrates were previously unknown; we demonstrated that four of these (RL2412, RL2961, pRL90140 and pRL100307) were secreted in a *prsD*-dependent manner, while another (RL0790) was subsequently shown to be secreted in complex (TY) growth medium in a *prsD*-dependent manner (A. Edwards and J. A Downie unpublished data). In addition to these predicted Type I substrates, we found that the presence of RL1580 (a predicted nucleoside diphosphate kinase) in the growth medium requires *prsD*. This suggests that the PrsDE system secretes at least thirteen proteins encoded by genes in diverse regions of the genome. This is a much broader specificity than typically observed with Type I secretion systems in other bacteria, where the secretion system is usually limited to the secretion of only one or a narrow range of closely-related substrates [83,84].

A role for Type I secretion in the symbiosis has been shown previously, since the nodulation signalling protein NodO is secreted via the PrsDE system, [11,12]. In addition, *prsD *mutants of *R.l*. bv *viciae *strain A34 and *R.l*. bv. *trifolii *strain TA1 form nodules that are infected but do not fix nitrogen [12,81]. This is not the case with *R.l*. bv *viciae *strain 3841 and the reason for the difference is not known, although a *prsD *mutant of *S. meliloti *also forms nitrogen-fixing nodules [15]. Although the PrsDE secretion system in *R.l*. bv. *viciae *3841 is not essential for nodulation, the proteins secreted by this system could nevertheless have an auxiliary function in nodulation as has already been demonstrated for NodO [5,6]. Several of the proteins whose secretion is *prsD*-dependent are predicted to be involved in surface attachment. This includes the *Rhizobium *adhering proteins RapA2, RapB and RapC identified previously [82], and RL2961 and pRL100309, which show similarity to eukaryotic cadherins. Cadherins promote calcium-dependent cell-cell adhesion in eukaryotic cells [63] indicating that RL2961 and pRL100309 could be involved in cellular aggregation, or attachment to roots an important part of legume infection. An extracellular calcium-binding protein of unknown sequence (rhicadhesin) has been identified as playing a role in rhizobial attachment to root hairs [85,86], but it is much smaller than RL2961 or pRL100309.

Two of the PrsDE-dependent secreted proteins are the glycosyl hydrolases PlyA and PlyB, which cleave the extracellular acidic exopolysaccharide [13]. A *plyA plyB *double mutant retained some glycosyl hydrolase activity, indicating that other extracellular glycosyl hyrolases were present [59]. It is possible that the other two predicted glycosyl hydrolases (PlyC and RL2412) could cleave rhizobial exopolysaccharides or could play a role in plant cell wall degradation similar to that observed previously in *R. l. bv. trifolii *[17].

RL0790 is predicted to be a secreted protease. The observation that it was present in complex medium (containing peptone), but not in minimal medium, suggests it may be induced if the bacteria are relying on peptides for growth. This is unlikely in the rhizosphere, but infection threads and senescent nodules are environments rich in proteins, and so it might be anticipated that RL0790 could contribute to growth of bacteria in infection threads or in senescing plant cells.

Nucleoside diphosphate kinase (NDK) was not predicted to be a Type I substrate by the bioinformatics approach, but was absent from the culture supernatant of the *prsD *mutant. Exported NDKs are not without precedent in mammalian pathogens; *P. aeruginosa, Vibrio cholerae *and *Mycobacterium tuberculosis *secrete NDKs [70,87-89], as do the parasitic nematodes *Trichinella spiralis *and *Haemonchus contortus *[90,91]. In *P. aeruginosa*, secretion of NDK into the culture supernatant has been shown to be dependent on the presence of the sequence DTEV near its C-terminus and it has been suggested that NDK secretion occurs via a Type I secretion system [70]. A very similar sequence (DTEI) is also present near the C-terminus of NDK from *R.l*. bv. *viciae *3841. Extracellular ATP plays a role in macrophage apoptosis as a response to pathogen attack. In *Arabidopsis thaliana*, auxin and ATP are both transported by the metabolite transporter MDR1. Removal of extracellular ATP leads to FB1-induced cell death in a number of plants including legumes [92]. Extracellular NDK may possibly have an effect on modulating these responses, but as with the other PrsDE-dependent secreted proteins its role is probably minor, since the *prsD *mutant lacking secretion of NDK does not appear to differ from the wild type in nodulation phenotype. A role for Type I secretion in the symbiosis has been shown previously because the nodulation signalling protein NodO is secreted via the PrsDE system, although this is not essential for nodulation [5,12]. In addition, *prsD *mutants of *R.l*. bv *viciae *strain A34 and *R.l*. bv. *trifolii *strain TA1 form nodules that are infected but do not fix nitrogen [12,81]. This is not the case with strain 3841 and the reason for the difference is not known, although a *prsD *mutant of *S. meliloti *also forms nitrogen-fixing nodules [15].

The predicted BltDE secretion system seems likely to be a bacteriocin/lantibiotic type exporter that secretes pRL90159 and pRl90161, large proteins encoded by genes upstream of the *bltDE *genes. Proteins similar to those encoded by *bltDE *are present in *R. etli *and a related system (Rzc) is involved in the export of medium bacteriocins in other rhizobia [79,80] implying a possible role for pRL90159 and pRl90161 as bacteriocin-like proteins. However, the sequences of pRL90159 and pRl90161 are more similar to hypothetical proteins from non-rhizobia such as *Nitrobacter spp*. than to proteins from other rhizobia

The other two predicted Type I secretion systems are enigmatic because we did not identify any secreted proteins that require *toaD *or *tobD *for their secretion, even though we made double and triple mutants (with *toaB*, *tobD *and *prsD*) to test for redundancy of function. This could mean that we have not identified appropriate growth conditions, the resolution of the protein gels is inadequate to identify the products (too low in amount or too small), or even that these are not protein secretion systems, but possibly secrete some other component. Genes similar to *toaD *are present in syntenic locations in *S. meliloti *(80% identity) [56] and *A. tumefaciens *(86% identity) [93]. A protein from *Bradyrhizobium japonicum *has similarity to TobD (52% identity). The genes surrounding the genes encoding these proteins also display similarity to those surrounding *toaD *and *tobD *in *R.l*. bv. *viciae *3841, respectively, suggesting synteny and likely orthology. It is evident that these genes are not essential for growth or for the symbiosis with the tested host legumes pea, broad bean and vetch.

### The TAT secretion system is involved in nodulation

The *R. l*. bv. *viciae *3841 *tatABC*-encoded secretion system genes are as expected from related bacteria such as *A. tumefaciens *[54] and *R. l*. bv. *viciae *UPM 791 [38]. The *tatC *mutant exhibited phenotypes expected from previous work in *A. tumefaciens *and *R. l*. bv. *viciae *UPM791 in that the *A. tumefaciens tatC *mutant was also shown to be largely immotile, lacking flagellar proteins in the culture supernatant and the *R. l*. bv. *viciae *UPM791 *tatC *mutant also formed white nodules on peas and did not fix nitrogen. However, while the *R. l*. bv. *viciae *UPM791 *tatC *mutant only formed small nodules, the nodules formed by a *R. l*. bv. *viciae *3841 *tatC *mutant were on average larger than those formed by the wild type. As in *R. l*. bv. *viciae *UPM791, the *R. l*. bv. *viciae *3841 *tatC *mutant did not progress beyond the infection zone in the nodule, but unlike in *R. l*. bv. *viciae *UPM791 [38], viable bacteria could be isolated from nodules formed by the *R. l*. bv. *viciae *3841 *tatC *mutant. The lack of growth and symbiotic nitrogen fixation in nodules could result from the inability of the *tatC *mutant to export to the periplasm electron-transport components required for respiration in the low oxygen environment in the nodule. Periplasmic proteins isolated from the *tatC *mutant and *R. l*. bv. *viciae *3841 grown in TY medium give very similar patterns when separated by SDS-PAGE, suggesting that most TAT substrate proteins are either not expressed under the observed conditions or are expressed at such a low level that they are not visible on the SDS-PAGE gel.

Another major difference between the wild type and the *tatC *mutant is the increased quantity of periplasmic proteins in the supernatant. A similar increased leakage of periplasmic contents into the extracellular space has been shown in *tatC *mutants of *E. coli *[94]. In this strain, outer membrane stability is compromised by the mislocalisation of two cell wall amidases, AmiA and AmiC, which are TAT substrates [95]. The AmiC protein from *R. l*. bv. *viciae *3841 (RL1742) is also predicted to be a TAT substrate, suggesting that the increased leakiness of the outer membrane is due to the mislocalisation of this protein.

### Identification of proteins from the culture supernatant

Previous studies on the extracellular proteome of rhizobia such as that by Suss et al. [96] have successfully used a proteomic approach to identify novel substrates of specific secretion systems, but have largely ignored proteins still present in the culture supernatant of the mutant studied. In this study, we show for the first time that many of the culture supernatant proteins identified by MALDI-Tof-MS are predicted to be periplasmic, most of them being similar to well known periplasmic proteins such as substrate binding proteins that are unlikely to function extracellularly. Type II protein secretion system secrete periplasmic proteins across the outer membrane in many Gram-negative bacteria, but such a system is absent from *R. l*. bv. *viciae *3841. Bands corresponding to the predicted periplasmic proteins in the culture supernatant were also observed in periplasmic preparations, arguing against a specific secretion mechanism and for unspecific leakage. Periplasmic proteins have also been identified in the culture supernatant of *H. pylori *[74], but the mechanism of secretion, if any, was also unknown. The absence of the cytoplasmic protein RhiA suggests leakage from the periplasm rather than cell lysis as the reason for the presence of periplasmic proteins in the culture supernatant. Periplasmic proteins are found in the culture supernatant of *R. l*. bv. *viciae *3841 at all stages of the cell cycle, and indeed are highly abundant during exponential growth. This indicates leakage from the periplasm during active growth, rather than cell death and lysis, as the likely origin of periplasmic proteins in the culture supernatant. Periplasmic proteins in the culture supernatant have previously been reported in *R. leguminosarum *RBL5523 [97]. The same study identified a mutant in which one of these proteins was absent from the culture supernatant and discussed the possible presence of a specific secretion system for this protein. To our knowledge, this is the only possible example of a periplasmic protein being secreted in rhizobia, and it seems to be limited to a single protein. Preliminary studies in our laboratory with an equivalent mutant in *R. l*. bv. *viciae *3841 did not reveal any difference in extracellular protein profile.

Only one Type III-like system was found in the genome sequence of *R. l*. bv. *viciae *3841, namely the flagellar biosynthetic cluster (Table 1). Although not classically regarded as a protein secretion system, the flagellar apparatus in *Salmonella *has also been shown to actively secrete proteins unrelated to flagellar biosynthesis [25], highlighting the possibility of secretion of non-flagellar proteins by the flagellar apparatus. It is not known whether *R. l*. bv. *viciae *3841 utilises the flagellum for protein secretion as well. Three components of the flagellar apparatus were identified in the culture supernatant. All three are part of the flagellum itself, and so are directly exposed to the extracellular medium. As the flagellum is easily sheared from the bacterial surface, flagellar proteins are common components of the culture supernatant in many bacteria [74-76,96]. Type IV secretion systems are involved in nodulation in M. loti R7A in a host-specific manner [20]. In contrast, the *virB6 *mutant of *R. l*. bv. *viciae *3841 was not affected in the nodulation process of any of the three host legumes tested in this study, showing that the putative Type IV protein secretion system in this strain is not essential for nodulation in these hosts.

## Conclusion

Although secreted proteins play a major role in the infection process of plant and animal pathogens and are also involved in successful nodulation in certain rhizobia-legume interactions, the identified homologs of known secretion systems (with the exception of TAT) do not appear to be essential for nodulation of pea plants by *R. l*. bv. *viciae *3841. The results do not rule out the possibility of a novel protein secretion mechanism operating in this strain, as the presence of a large number of proteins in the culture supernatant remains unexplained. As these proteins are predicted to be periplasmic and indeed also abundant in periplasmic preparations, unspecific leakage from the periplasm could also account for these. Protein secretion in *R. l*. bv. *viciae *3841 is furthermore distinct from that of many other Gram-negative bacteria in that the main pathway of protein secretion appears to be a Type I secretion system, PrsDE. This system is predicted to secrete at least 13 very dissimilar proteins of widely varied molecular weights. This is in direct contrast to the very narrow range of substrates recognised by most other Type I systems. The PrsDE system is conserved in other rhizobia and is required for nodulation in some of these, but not in *R. l*. bv. *viciae *3841. The predicted functions of many of the PrsDE-dependent proteins are nevertheless consistent with auxiliary function in nodulation such as attachment, cell wall degradation, escape from senescent nodules or the modulation of the extracellular ATP pool.

## Methods

### Media and growth conditions

Strains of *Rhizobium *were grown at 28°C in Tryptone-Yeast (TY) medium [98] or Y [99] medium supplemented with succinate or mannitol as indicated in the text. Solid media contained 1% agar unless stated otherwise. Antibiotics were used at the following concentrations: ampicillin 400 μg ml^-1^; gentamicin 20 μg ml^-1^; kanamycin μg ml^-1^; spectinomycin 400 μg ml^-1^; streptomycin 400 μg ml^-1^. PGase and glycanase activity were assayed on plates as described by Finnie et al. [12]. Motility was assayed by observing the absence or presence of swarming on 0.2% agar TY plates.

### Nodulation assay

Nodulation assays on *Pisum sativum *cv. Frisson and *Vicia faba *cv. The Sutton were done as described previously for peas by Beynon et al. [100]. Briefly, seeds were rinsed with 70% ethanol, surface-sterilised by treatment with 5% sodium hypochlorite for 10 minutes and germinated on agar medium in the dark. *Vicia hirsuta *seeds were scarified with sulphuric acid and germinated on agar slants as described by Knight et al. [101].

### Protein electrophoresis

Discontinuous sodium dodecyl sulphate polyacrylamide gel electrophoresis (SDS-PAGE) was used to separate denatured proteins according to molecular weight [102]. The discontinuous system consisted of a stacking gel (4% (w/v) acrylamide, pH 6.8) and a separating gel (12% (w/v) acrylamide, pH 8.8). The gels were cast and run in the vertical Biorad MiniProtean II system. Samples were prepared by adding an equal volume of 2 × SDS sample buffer and incubating for 10 min in a boiling water bath. Gels were run at 25 mA per gel using 50 mM Tris/glycine pH 8.6, 0.1% (w/v) SDS as running buffer.

### Preparation of culture supernatant proteins

Cells grown in 100 ml of liquid Y mannitol medium for four days until stationary phase were pelleted by centrifugation for 45 min at 15,000 × *g *at 4°C. 20% (w/v) trichloroacetic acid (TCA) was added to the supernatant to a final concentration of 5% (w/v). After 30 min incubation on ice, the supernatant proteins were pelleted by centrifugation for 1 h at 15,000 × *g *at 4°C. The pellet was washed twice with 80% (v/v) acetone, 50 mM Tris/HCl pH 8.0 to remove residual TCA and air-dried prior to resuspension in SDS-PAGE sample buffer. Other methods to precipitate culture supernatant such as acetone precipitation, ammonium sulphate precipitation or lyophilisation of the culture supernatant were found to be ineffective for this strain due to the presence of large amounts of exopolysaccharide that were co-precipitated by these methods.

### Generation of mutants

Mutants are listed in Table 4 and all except *toaD *and *tatC *were identified by PCR from an arrayed random Tn5-insertion library using gene- and Tn*5*-specific primers. The locations of insertion of Tn5 were confirmed by DNA sequencing. Strains carrying an insertion in two of the genes of interest, tatC and toad, were not present in this random library and were therefore constructed by double homologous recombination. For the construction of a *toaD *mutant, a 2 kb fragment carrying the *toaD *gene was cloned into pJQ200SK [103] and a spectinomycin resistance cassette ligated into the unique *Nco*I site within *toaD*. This construct was then recombined into the genome by a double homologous recombination. The *tatC *mutant was generated using a similar approach to that used to inactivate *toaD*. PCR and DNA sequencing were used to verify that all insertions interrupted the gene of interest.

**Table 4:** Strains used in this work

**Strain**	**Description**	**Reference**
3841	*R*.*l*. bv. *viciae *wild-type Str^R^	[108]
A895	3841 carrying *prsD*::Tn5	This study
A896	3841 carrying *tobD*::Tn5	This study
A897	3841 carrying *virB6*::Tn5	This study
A898	*prsD*::Tn5(gent): A895 containing gentamicin cassette replacing Tn5-neomycin resistance	This study
A903	*prsD*::Tn5(gent), *tobD*::Tn5; made by transduction of A896 with phage from A898	This study
A911	*prsD*::Tn5(gent), *toaD*::spec; made by transduction of A898 with phage from A913	This study
A912	*prsD*::Tn5(gent), *tobD*::Tn5, *toaD*::spec; made by transduction of A903 with phage from A913	This study
A913	3841 carrying *toaD*::spec	This study
A1010	3841 carrying *autA*::Tn5	This study
A1011	3841 carrying *autB*::Tn5	This study
A1012	3841 carrying *autC*::Tn5	This study
A1052	3841 carrying *tatC*::spec	This study

To avoid the problem of possible redundancy between the different members of Type I protein secretion systems, a *prsD*/*toaD*/*tobD *triple mutant and all double combinations were generated. To achieve this, the kanamycin resistance in the *prsD *mutant A895 was exchanged for gentamicin resistance by homologous recombination within the Tn*5 *sequence using plasmid pJQ175 [104], resulting in strain A898. Gentamicin resistance from this *prsD *mutant (A898) and spectinomycin resistance from the *toaD *mutant (A913) were then transduced consecutively into the kanamycin resistant *tobD *mutant (A896) to produce the *prsD*/*toaD*/*tobD *triple mutant A912. The double mutants *toaD/prsD *(A911) and *tobD/prsD *(A903) were constructed similarly. The mutants were checked by PCR and DNA sequencing. Although also part of a putative Type I secretion system, the BltD protein clearly belonged to a different class of proteins than the other three Type I ABC protein exporters. Therefore double or triple mutants protein exporter mutants carrying the *bltD *mutation were not made.

### Analysis of sequence data

Nucleotide sequence data was curated and annotated using the CloneManager or Artemis software packages. Versions of BLAST [53] were used to carry out similarity searches in various databases. Sequences were aligned using ClustalX 1.8 [105], TreeView [106] was used to visualise dendrograms and phylograms. GEP signal sequences were predicted using the programs PrediSi and SignalP 3.0, TATFIND and TATP 1.0 were used for the prediction of TAT substrates. The RL and pRL numbers refer to gene identifiers used in the genome sequence of *R.l*. bv. *viciae *strain 3841 [107].

### MALDI-ToF MS

The proteins contained in the gel slices (approx. 2 × 8 mm) were submitted to tryptic digest and MALDI ToF MS fingerprinting. The MS data were queried against a database containing the predicted *R. l*. bv. *viciae *3841 protein sequences using the MASCOT software. Proteins achieving a MOWSE score of more than 51 (representing more than 95% confidence) were regarded as positively identified. Most proteins were identified with more than 99% confidence. All MASCOT searches were carried out on the complete database without molecular weight constraints, but the theoretical molecular weights of the predicted proteins all lay within 15 kDa of the molecular weight observed on the gel. Restricting the upper molecular weight limit adds additional data to the database search parameters and would result in even higher MOWSE scores, especially for relatively small proteins. As the molecular weight of many proteins can be estimated from an SDS-PAGE gel, this would be a legitimate constraint under the assumption that there is no major posttranslational processing. The MOWSE score for any individual proteins in a protein mixture is reduced, as many of the fragments in a mixture would remain unmatched to any single protein. For this reason, proteins identified from band 12 (which comes from a region of the gel with many closely-spaced protein bands) tend to have lower MOWSE scores than proteins matched to clear single bands. Two proteins from band 12 (RL0713 and RL0489) actually achieve MOWSE scores below the statistical cutoff at 51. Considering their molecular weights and the fact that they clearly come from a mixed band, they were nevertheless included. Removing fragments from the positively identified proteins pRL100451 and RL0196 from the band 12 dataset lifts the MOWSE score above the cutoff. This is not the case for low scoring proteins in the other datasets.

## Authors' contributions

MK performed the bioinformatic analysis, carried out the experiments and drafted the manuscript jointly with JAD, who coordinated the study. Both authors have read and approved the final manuscript.
